# A Multiple-Stimuli-Responsive Amphiphilic Copolymer for Antifouling and Antibacterial Functionality via a “Resistance–Kill–Release” Mechanism

**DOI:** 10.3390/molecules27165059

**Published:** 2022-08-09

**Authors:** Xiaohan Liao, Kai Niu, Feng Liu, Yongming Zhang

**Affiliations:** School of Chemistry and Chemical Engineering, Shanghai Jiao Tong University, Shanghai 200240, China

**Keywords:** stimuli-responsive, amphiphilic polymers, host–guest interactions, antifouling, antibacterial

## Abstract

In recent years, polymers with stimuli-responsive properties have been increasingly reported on due to their diverse applications. However, most of the studies have only focused on the performance of polymers under specific scenarios. The laws of changes in the properties in response to various external stimuli have been less systematically and quantitatively studied. In this paper, we prepared an amphiphilic polymer (PadaMX and PAdaM3QA−X) with temperature-, pH-, ion-, and β-cyclodextrin (β-CD)-responsive properties. According to the cloud point tested by the UV-Vis method, the lower critical soluble temperature (LCST) of PAdaM3QA−10% was more sensitive to a change in pH and less sensitive to a change in ions compared with PadaM3 due to quaternized side chains with a stronger intramolecular mutual repulsion. We then fabricated the coatings with responsive properties by immobilizing the adamantyl groups on β-CD-modified surfaces. The hydrophilicity of the coatings was improved after quaternization, as proven by the water contact angle (WCA) measurement. The antifouling and antibacterial performance was further evaluated via the fluorescence intensity of bovine serum albumin (BSA) adsorbed on the surfaces and the spread plate method. A 78.4% BSA desorption rate and a 96.8% sterilization rate were achieved by the PAdaM3QA−10% coating. In summary, this work prepared a multiple-stimuli-responsive amphiphilic copolymer for antifouling and antibacterial functionality via a “resistance–kill–release” mechanism.

## 1. Introduction

In recent years, novel functional polymers that can respond to changes in external environmental conditions have attracted extensive interest in academia and industry. Such smart polymers are defined as stimuli-responsive polymers. Stimuli-responsive polymers can respond to subtle physical or chemical changes in the external environment, including the formation or destruction of chemical bonds, changes in solubility, or changes in the molecular configuration or conformation [1,2,3,4,5]. External environmental stimuli include the temperature, pH, ionic strength, light, and active molecules [6,7,8,9]. From the perspective of molecular design, polymers can be designed to respond to only a single stimulus; two or more stimuli-responsive groups can be introduced into polymers with different topologies to achieve multiple-stimuli-responsive performances. From the perspective of material design, cross-linked networks, self-assembled structures, nanomaterials, and composite materials can be further constructed on the basis of stimuli-responsive polymers [10,11,12]. For example, copolymer hydrogels consisting of poly (dimethylaminoethyl methacrylate) (PDMAEMA) and 3-acrylamidophenylboronic acid have been reported to be pH-, temperature-, and glucose-responsive; that is, the PDMAEMA moiety and the acid moiety were pH/temperature- and glucose-responsive, respectively. The hydrogels exhibited activity in response to glucose under physiological conditions and the size of the gel network could be tuned by changing the temperature and pH of the environment [13]. Homopolymers such as electro-responsive polyelectrolyte can be grafted onto nanofluidic channels to control the flow rate by performing a stretch–collapse transition under external electric fields [14]. Hence, multiple-stimuli-responsive polymers are widely applied in chromatography [15,16], optoelectronics [17,18], drug delivery [19,20], and biosensors [21,22]. However, most of the research focuses only on the changes of the structures and the performance of polymers before and after measuring their responsiveness under specific conditions and only a few studies have systematically and quantitatively studied the laws and trends of polymer properties changing with external stimuli.

Stimuli-responsive polymers are often immobilized as “polymer brushes” on substrate surfaces, which can be solid [23,24], colloidal [25], or polymeric membrane surfaces [26], by either physical adsorption or chemical bonding. Physical adsorption is relatively simple to operate, but the coatings fabricated via this method are prone to failure due to the lack of a strong interaction between the substrate surfaces and the coatings [27,28]. Substrate surfaces modified with stimuli-responsive polymers via strong covalent bonds or reversible supramolecular interactions have more stable properties. The “grafting-from” concept has been applied to fabricate high-density polymer brushes; for example, ABA-type block polymer brush hollow capsules have been prepared by surface-initiated single-electron transfer living radical polymerization (SET-LRP) from an initiation site on silica surfaces that served as a template [29]. Another concept, “grafting-to”, is usually realized in the form of supramolecular interactions between precisely designed molecules. In a previous study, a dextran layer bearing adamantyl groups was immobilized on β-cyclodextrin (β-CD)-modified porous silica particles by a host–guest interaction to form inclusion complexes [30].

The most common applications for stimuli-responsive polymer brushes are as antifouling and antibacterial coatings [31,32]. As temperature and pH are significant indicators of the internal environment, temperature- or pH-responsive polymers are widely used to detect changes in the internal environment and to respond accordingly [33,34]. Based on the different antifouling mechanisms of polymers, there are fouling resistance, fouling release, and fouling degradation coatings [31]. In recent years, antifouling and antibacterial coatings that possess two or more antifouling mechanisms have emerged as promising solutions for bacterial infection and contamination problems [35,36]. For example, smart “resistance–kill–release” coatings can resist the adhesion of contaminants due to their hydrophilicity and can switch functionality between bacteria killing and bacteria/fouling release under a proper stimulus. These coatings combine the advantages of the biocidal activity of cationic polymers [37], the fouling resistance of hydrophilic polymers [38], and the fouling release properties of stimuli-responsive polymers in a switchable way. Such copolymers can effectively prevent the adhesion of bacteria/fouling and the subsequent formation of biofilms on the surface of materials. For medical implants and devices, reducing the bacterial attachment can improve the device life and reduce clinical complications caused by bacteria. For food processing and packaging materials, reducing the accumulation of contaminants can improve the food quality. For marine equipment, reducing the contamination on surfaces can reduce the operating and maintenance costs [39,40]. Therefore, significant attention has been paid to the development of such coatings to reduce the initial attachment of contaminants, to kill bacteria, and to prevent any subsequent biofilm formation.

The aim of this work was to construct a multiple-stimuli-responsive polymer via a simple and efficient preparation process that could respond to multiple main indicators of the internal environment. Herein, dimethylaminoethyl methacrylate (DMAEMA), which is responsive to temperature, pH, and ionic strength, was selected and a hydrophobic monomer, adamantyl methyl methacrylate (AdaMMA), which is the guest molecule in the host–guest interaction with β-cyclodextrin (β-CD), was introduced to prepare the amphiphilic copolymer (PAdaMX). The amphiphilic copolymer was then further quaternized (PAdaM3QA−X) and immobilized on a β-CD-modified substrate to fabricate “resistance–kill–release” antifouling and antibacterial surfaces. In addition, the property changes of the products to stimulations of different intensities were systematically and quantitatively studied and discussed.

## 2. Results and Discussion

### 2.1. Synthesis and Characterization of AdaMMA, PAdaMX, and PAdaM3QA−X

**Figure 1:**
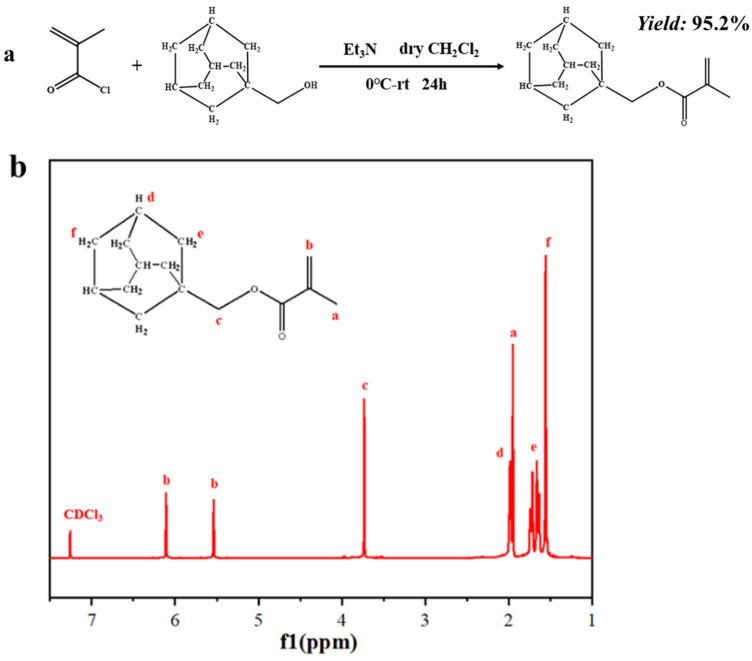
(**a**) The schematic illustration of the synthesis of AdaMMA; (**b**) ^1^H-NMR spectra of AdaMMA (in deuterochloroform).

Triethylamine and 1-adamantane methanol were dissolved in 200 mL anhydrous DCM in a glove box, followed by the addition of MAC at 0 °C. The reaction mixture was allowed to warm slowly to room temperature and stirred for 24 h. The insoluble salts were filtered and the filtrate was then extracted with a saturated NaCl solution, 1 M HCl, 1 M NaHCO_3_, and saturated NaCl solution. The organic phase was dried over anhydrous Na_2_SO_4_ and filtered; the solvent was removed on a rotary evaporator to obtain a colorless viscous liquid that solidified in the refrigerator to a white solid (yield: 95.2%). The schematic illustration of the synthesis of AdaMMA and the ^1^H-NMR spectra are displayed in Figure 1a,b.

**Figure 2:**
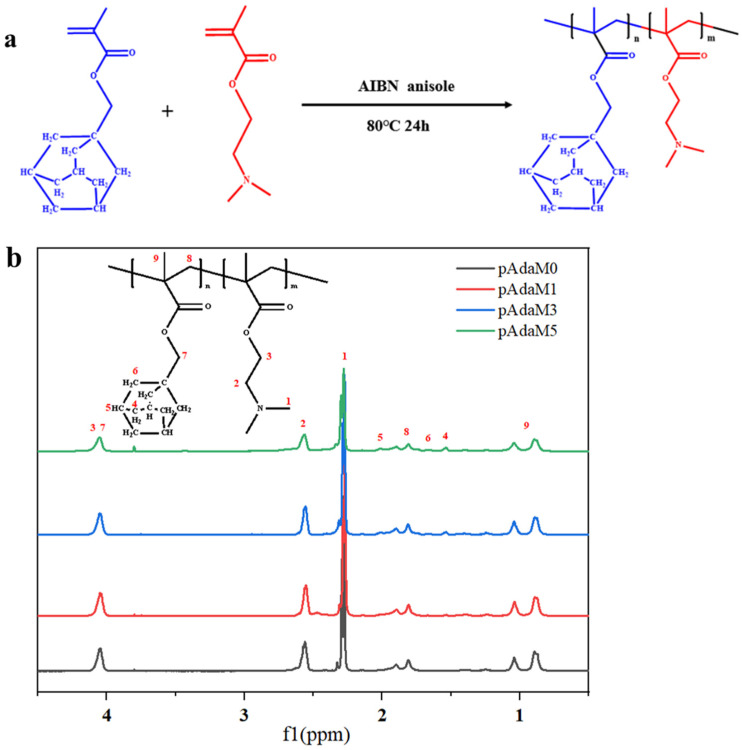
(**a**) The schematic illustration of the synthesis of PAdaMX; (**b**) ^1^H-NMR spectra of PAdaMX (in deuterochloroform).

PAdaMX was prepared by changing the feeding ratio of DMAEMA and AdaMMA whilst maintaining the same amount of AIBN (Table 1). The monomer consumption was calculated by the integration of the signal of the DMAEMA and AdaMMA vinyl groups (CHH=C CH_3_, 6.11 ppm, or 5.56 ppm) against the internal standard (anisole, *o,p* Ar-H, 6.91 ppm) [36]. A certain amount of DMAEMA, AdaMMA, and AIBN was dissolved in anisole. The flask was immersed in an oil bath at 80 °C and the reaction was stopped after 24 h. The product was purified by 3 precipitations from n-hexane and dried under a vacuum for 12 h at 40 °C. The schematic illustration of the synthesis of PAdaMX and the ^1^H-NMR spectra is displayed in Figure 2a,b.

**Figure 3:**
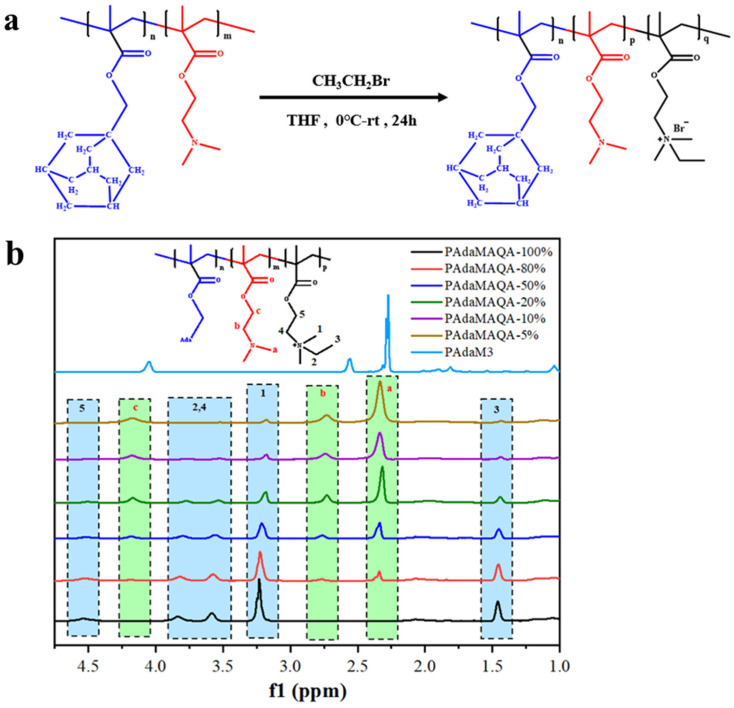
(**a**) The schematic illustration of the synthesis of PAdaM3QA with different degrees of quaternization; (**b**) ^1^H-NMR spectra of PAdaM3QA−X (in deuteroxide).

PAdaM3QA−X with a different degree of quaternization was synthesized by changing the amount of bromoethane (Table 2). PAdaM3 was dissolved in THF in a 50 mL flask and sealed in a glove box, followed by the addition of different volumes of bromoethane at 0 °C. The reaction mixture was allowed to slowly warm to room temperature and stirred for 24 h. The product was purified by 3 precipitations from n-hexane and dried under a vacuum for 12 h at 40 °C. The PAdaM3QA−100% sample was precipitated after the reaction was completed, washed with n-hexane 3 times and dried under a vacuum for 12 h at 40 °C. The schematic illustration of the synthesis of PAdaM3QA with different degrees of quaternization and the ^1^H-NMR spectra are displayed in Figure 3a,b.

All 0.25 wt% polymer solutions were prepared by dissolving 0.0525 g polymers in 20 mL deionized water.

### 2.2. Temperature-Responsive Properties of PAdaMX and PAdaM3-X

**Figure 4:**
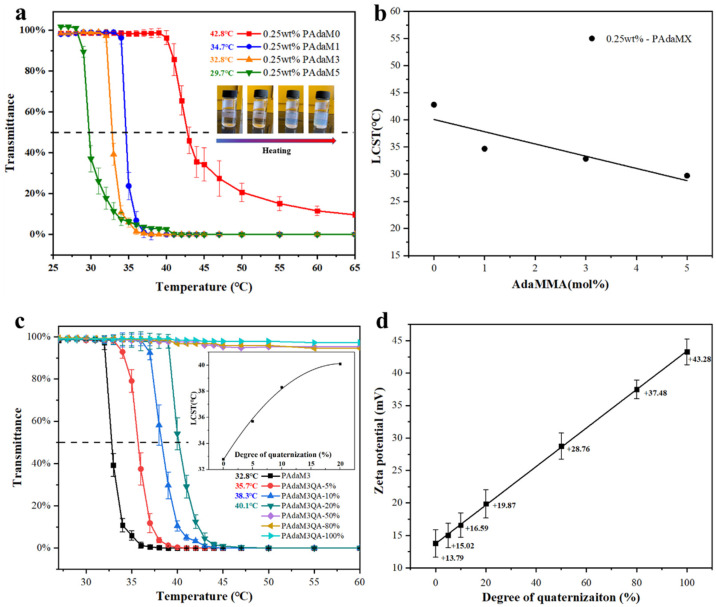
(**a**) The transmittance of 0.25 wt% PAdaMX solution; (**b**) the effect of AdaMMA addition on LCST of PAdaMX solution; (**c**) the transmittance of PAdaM3QA−X solution; (**d**) the zeta potential of PAdaM3QA−X solution with different degrees of quaternization.

Figure 4a shows the transmittance of the PAdaMX aqueous solution at different temperatures, indicating that an obvious lower critical soluble temperature (LCST) could be observed in the solution. The LCSTs of the 0.25 wt% PAdaMX solution with different [DMAEMA]:[AdaMMA] ratios were 42.8 °C, 34.7 °C, 32.8 °C, and 29.7 °C, respectively. As shown in Figure 4b, under the same mass fraction, the LCST of the PAdaMX solution decreased approximately linearly with the addition of AdaMMA. This was attributed to the introduction of the strong hydrophobic monomer AdaMMA, resulting in a reduction in the overall hydrophilic/hydrophobic group ratio of the copolymer. As a result, the number of hydrogen bonds formed between the copolymer and the water molecules was reduced and it took less energy to break those hydrogen bonds [41].

PAdaM3 was chosen for a further modification to the polymers with different degrees of quaternization. Figure 4c presents the transmittance of the PAdaM3QA−X solutions with different degrees of quaternization under different temperatures. It can be seen from the figure that the LCSTs of the PAdaM3QA−X solutions with 5%, 10%, and 20% quaternization degrees were 35.7 °C, 38.3 °C, and 40.1 °C, respectively; these were higher than those of PAdaM3, but still showed temperature responsiveness and the LCST increased with the increase in quaternization degree. However, the LCST diminished when the degree of quaternization increased to more than 20%. As displayed in Figure 4d, the zeta potential of the PAdaM3Q-X solutions was tested to evaluate the contribution of quaternization to the surface charge and dispersibility of PAdaM3Q-X. We observed that the zeta potential was proportional to the degree of quaternization. This illustrated that the introduction of quaternary ammonium moiety could significantly increase the charge of PAdaM3QA−X in the solution, thereby improving the hydrophilicity of the polymer [42]. In this case, a higher temperature was required to destroy the interaction between PAdaM3QA−X and the water molecules, resulting in an increase in the LCST. When the degree of quaternization increased to more than 20%, PAdaM3QA−X was too hydrophilic to respond to the temperature changes.

### 2.3. pH-Responsive Properties of PAdaM3 and PAdaM3QA−10%

To further investigate the effect of pH on the temperature-responsive properties of the PAdaMX and PAdaM3QA−X solutions, PAdaM3 and PAdaM3QA−10% were selected for further studies in order to avoid the inaccuracy of the LCST caused by a wide temperature variation range (the range where transmittance decreased from 100% to approximately 0%) as the narrowest variation ranges were observed in the PAdaM3 and PAdaM3QA−10% solutions among the PAdaMX and PAdaM3QA−X solutions, as shown in Figure 4a,c. Therefore, PAdaM3 and PAdaM3QA−10% were prepared into 0.25 wt% aqueous solutions with pH = 2, 4, 6, 8, 9, 10, 11, 12, and 13 for UV-Vis testing.

**Figure 5:**
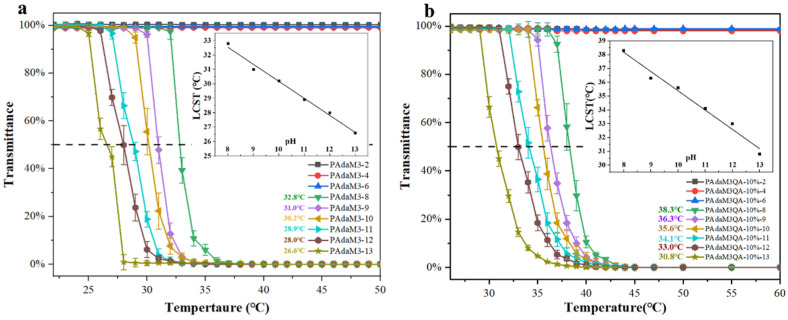

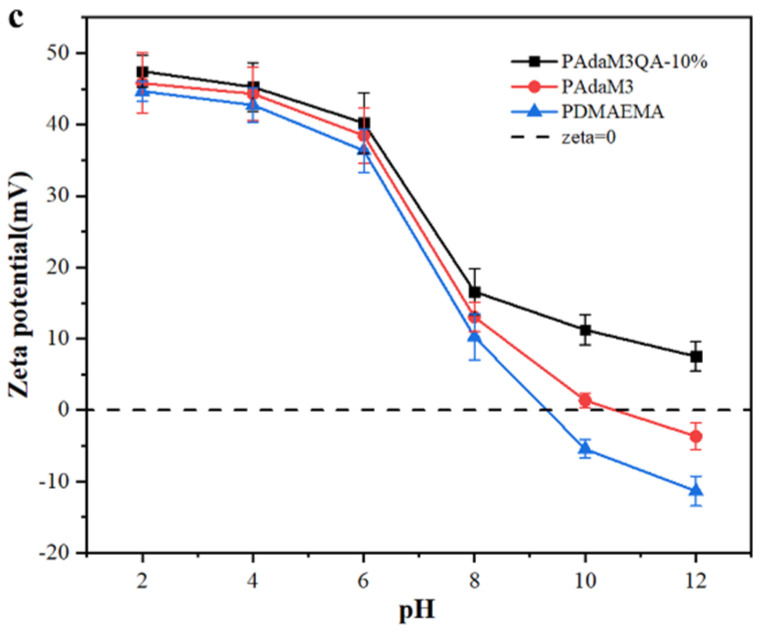
(**a**) The transmittance of PAdaM3 solution at different pHs; (**b**) the transmittance of PAdaM3QA−10% solution at different pHs; (**c**) the zeta potential of PAdaM3QA−10%, PAdaM3, and PAdaM0 solutions at different pHs.

Figure 5a,b demonstrates that the LCSTs of the PAdaM3 and PAdaM3QA−10% solutions both decreased with an increase in the pH when the pH was greater than or equal to 8. However, when the pH was less than 8, no LCST was observed as the temperature increased. This phenomenon was due to the protonation of the tertiary amine groups (-N(CH_3_)_2_) of the DMAEMA moiety in an acidic solution [43]. The degree of protonation (β) of the polymer at a given pH could be calculated by the Henderson–Hasselbalch formula [44]:

pH = pK_a_ + log[(1 − β)/β] (1)

When the pH < pK_a_ (≈7.4 at room temperature for DMAEMA), the tertiary amine group with strong nucleophilicity in the side chain was highly protonated to form a quaternized group (e.g., β = 96.2% when the pH was 6; β = 99.6% when the pH was 5). The hydrogen bonding between the quaternary ammonium group and the water was much stronger than that of the tertiary amine group, so the side chain of the polymer tended to stretch into a random coil conformation in an aqueous solution, resulting in the observation that the change in temperature had no obvious effect on the shrinkage of the polymer segments; macroscopically, there was no LCST observed. However, when the pH > pK_a_, the side chains maintained the tertiary amine moiety and the degree of protonation abruptly decreased as the temperature increased (e.g., β = 20.1% when the pH was 8; β = 2.5% when the pH was 9). Therefore, the hydrogen bond between the side chains and the water was destroyed as the temperature increased. Moreover, with the domination of the hydrophobic bonds, the polymer chains were more likely to intertwine and consequently aggregate; the LCST appeared macroscopically.

By fitting the relationship between the pH and the LCST of the PAdaM3 and PAdaM3QA−10% solutions (inset of Figure 5a,b), it could be seen that the LCST decreased approximately linearly with an increase in the pH. The fitting formulae were:

LCST_PAdaM3_ = −1.18·pH + 41.97 (°C) (2)

LCST_PAdaM3QA−10%_ = −1.40·pH + 49.35 (°C) (3)

where the slope represented the sensitivity of the LCST to the pH. PAdaM3QA−10% was more sensitive to the changes in pH than PAdaM3, according to the formulae. This was because the effect of electrostatic screening on the LCST was stronger than that caused by the decrease in the protonation degree as the pH (NaOH addition) increased. Specifically, the presence of ions from NaOH weakened the intramolecular mutual repulsion of the side chains, making them collapse easier. The degree of protonation decreased from only 2.5% to almost 0% when the pH increased from 9 to 13. In this case, the quaternary amine group was more sensitive to the change in pH compared with the tertiary amine group in the alkaline solution.

As depicted in Figure 5c, the PAdaM3QA−10% solution remained positively charged under alkaline conditions; the zeta potential of PAdaM3 and PAdaM0 decreased significantly more due to the stronger deprotonation of the tertiary amine group, which was the reason why PAdaM3 was more likely to aggregate and had a lower LCST than PAdaM3QA−10% at the same pH.

### 2.4. Ion-Responsive Properties of PAdaM3 and PAdaM3QA−10%

In order to investigate the ion responsiveness of PAdaM3 and PAdaM3QA−10%, different masses of NaCl, NaBr, KI, and NH_4_Cl were added to the polymer solutions and the change in transmittance of the solution with the temperature was tested.

**Figure 6:**
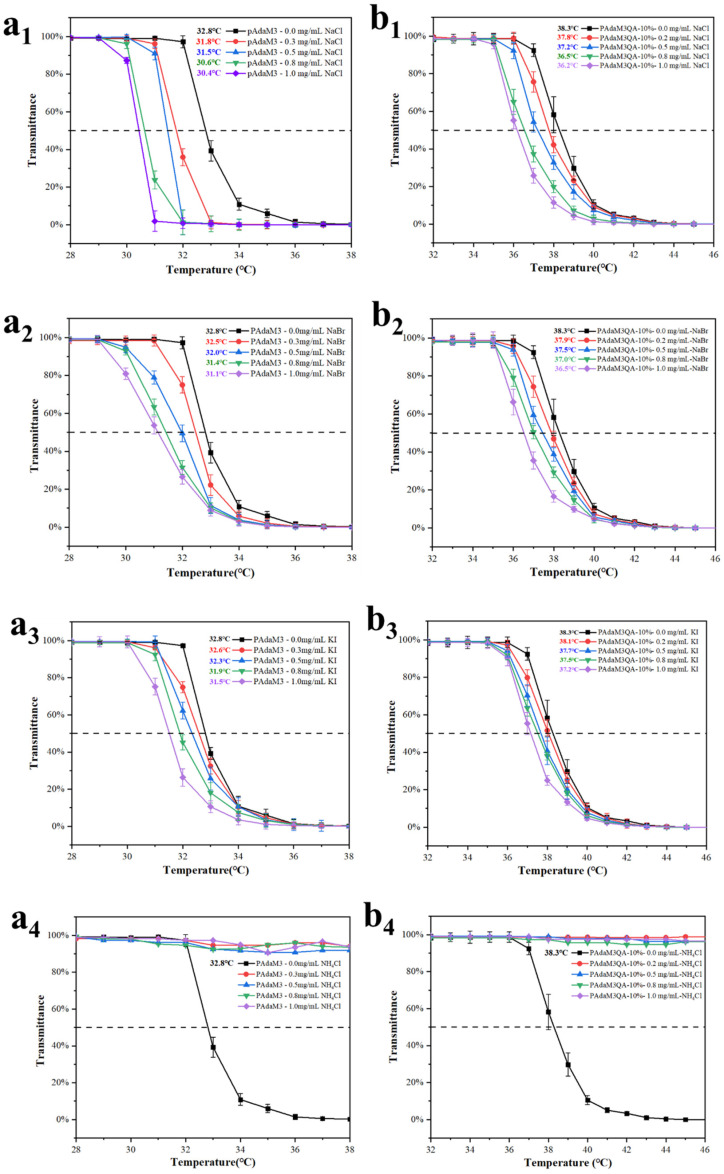
The effect of salt addition on the transmittance of PAdaM3 solution: (**a_1_**) NaCl; (**a_2_**) NaBr; (**a_3_**) KI; (**a_4_**) NH_4_Cl. The effect of salt addition on the transmittance of PAdaM3QA−10% solution: (**b_1_**) NaCl; (**b_2_**) NaBr; (**b_3_**) KI; (**b_4_**) NH_4_Cl.

The addition of NaCl, NaBr, and KI all decreased the LCSTs of the solutions (Figure 6(a_1_–a_3_,b_1_–b_3_)), which was due to the electrostatic screening mentioned in Section 2.2. It should be noted that the disappearance of the LCST occurred after NH_4_Cl was added (Figure 6(a_4_,b_4_)). The main reason was that NH_4_^+^ was hydrolyzed in the aqueous solution and made the solution weakly acidic, leading to the protonation of the DMAEMA moiety [45]. As a result, the hydrophilicity of the polymer greatly improved, causing the LCST to disappear.

**Figure 7:**
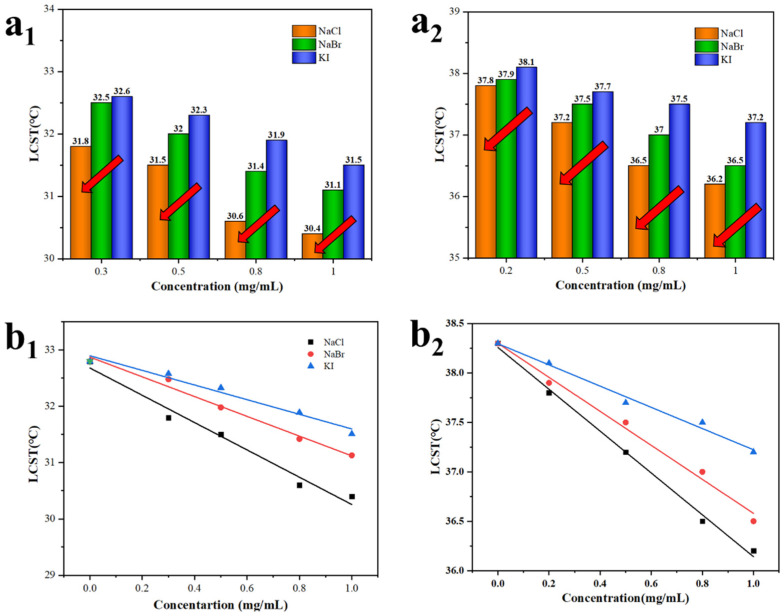
The effect of different ions on the LCST of (**a_1_**) PAdaM3 and (**a_2_**) PAdaM3QA−10% solutions; the fitting curve between the ion concentration and the LCST of (**b_1_**) PAdaM3 and (**b_2_**) PAdaM3QA−10% solutions.

Interestingly, the influence on the LCST decreased in the order of NaCl, NaBr, and KI under the same ion concentration (Figure 7(a_1_,a_2_)). This could be explained by the combined effect of ionic binding and electrostatic screening. The anion could directly bind to the positively charged protonated tertiary amine group in the solution, which enhanced the extension of the polymer chains in the solution and thereby increased the LCST of the solution. This process partially offset the electrostatic screening [46]. Moreover, although the valence state of the ions was the same, the different binding constants could also make the solution LCST decrease differently under the same concentration. However, as the concentration of the ion increased, the ion binding was saturated and the LCST decreased due to the increase in the electrostatic screening effect. The ability of the anions to decrease the LCST of the solution was similar to the Hoffman sequence [47,48]; that is, Cl^−^ > Br^−^ > I^−^.

The parameters of the fitting curve (Figure 7(b_1_,b_2_)) for the ion concentration and the LCST of PAdaM3 and PAdaM3QA−10% are shown in Table 3. The results showed that the LCST of the solution was linearly negatively related to the ion concentration and all of them had a high correlation coefficient (*R*^2^). It is notable that PAdaM3QA−10% was less sensitive to the change in ion concentration than PAdaM3, according to the slope (*k*) of the fitting curves; this was ascribed to the more intensive intramolecular mutual repulsion after quaternization. Thus, the polymer segment was more stretched in the solution, offsetting the electrostatic screening to an extent, so the sensitivity of PAdaM3QA−10% to the ion concentration was lower than that of PAdaM3.

### 2.5. β-CD-Responsive Properties of PAdaM3 and PAdaM3QA−10%

β-CD has a three-dimensional conical cavity structure that is composed of a hydrophilic rim and a hydrophobic cavity. It usually serves as a host molecule to form a supramolecular complex with guest molecules. The adamantyl group of PAdaM3 and PAdaM3QA−10% was a typical guest molecule that could be recognized by β-CD to form a stable complex in an aqueous solution.

**Figure 8:**
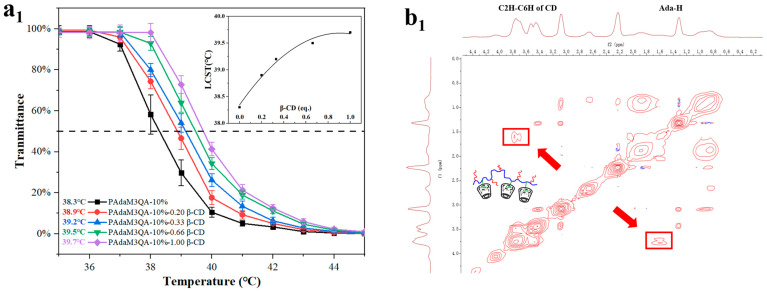

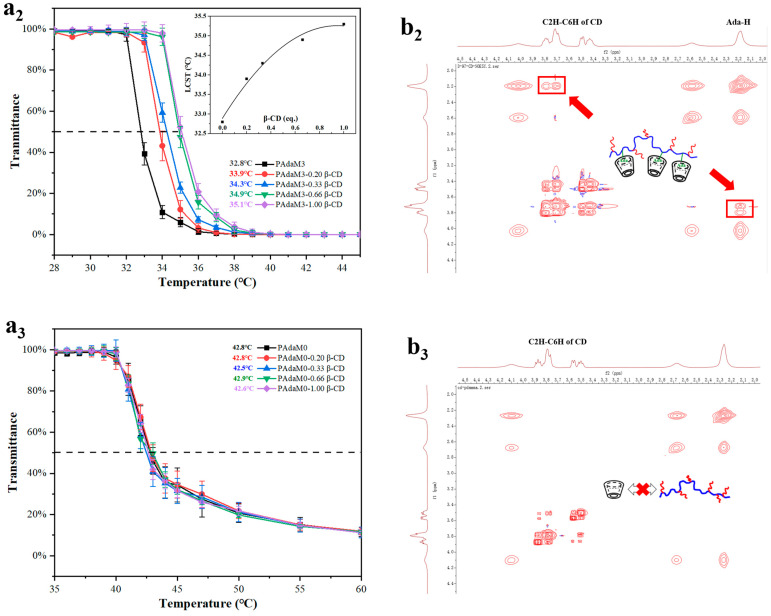
The effect of β-CD on the LCST of (**a_1_**) PAdaM3QA−10%, (**a_2_**) PadaM3, and (**a_3_**) PAdaM0 solutions; partial 2D NMR NOESY spectra of the mixture of β-CD and (**b_1_**) PAdaM3QA−10%, (**b_2_**) PAdaM3, and (**b_3_**) PAdaM0 in water at 30 °C.

Figure 8(a_1_–a_3_) depict the change in LCST after the addition of different masses of β-CD. Taking the fitting curves into account (inset of Figure 8(a_1_,a_2_)), the results demonstrated that the LCST of the PAdaM0 solution had no significant change when β-CD was added, but the LCST of the PAdaM3 and PAdaM3QA−10% solutions displayed an upward trend with the increasing addition of β-CD. We speculated that β-CD had a good host–guest interaction with the adamantyl group in PAdaM3 and PAdaM3QA−10% in aqueous solutions so that the hydrophobic adamantane moiety was wrapped in the inner cavity of β-CD; the hydrophilic outer rim of β-CD helped to improve the hydrophilicity of the polymers, which increased the LCST of the solution [49,50]. However, there was no adamantyl group in PAdaM0, which indicated that adding β-CD had no obvious effect on the LCST of the PAdaM0 solution. The host–guest interactions were further proven by the results of 2D-NMR NOESY. There were cross-peaks of interaction between PAdaM3/PAdaM3QA−10% and β-CD in the mixed solution of PAdaM3/PAdaM3QA−10% and β-CD (Figure 8(b_1_,b_2_)); however, no cross-peak could be found in the spectraof the PAdaM0 and β-CD mixed solution (Figure 8(b_3_)).

Surprisingly, we found that the solution LCST did not linearly increase with β-CD, but showed a gradually decreasing rate of ascent instead. This resulted from the limited binding sites of the adamantyl side groups on PAdaM3/PAdaM3QA−10%. When the binding of β-CD and adamantyl groups was close to saturation, the effect of increasing the addition of β-CD on the LCST gradually decreased.

### 2.6. Wetability of PAdaM3 and PAdaM3QA−10% Coatings

**Figure 9:**
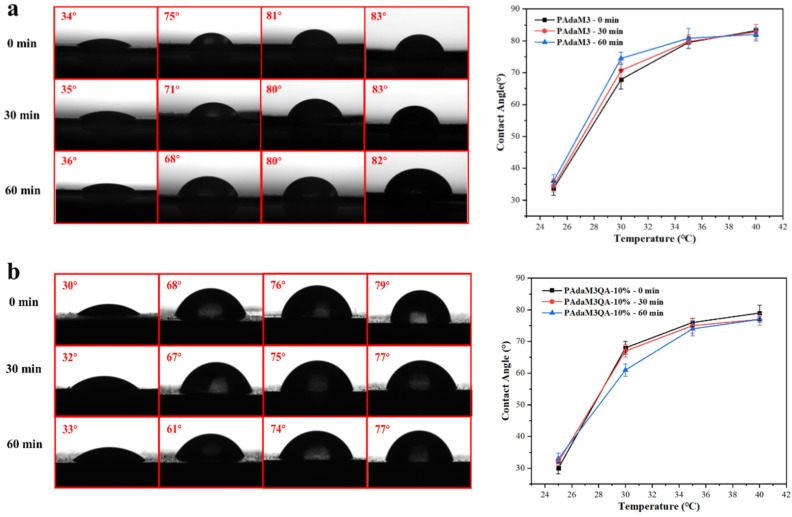
(**a**) The images and curves of WCA of PAdaM3 coating; (**b**) the images and curves of WCA of PAdaM3QA−10% coating.

The water contact angle (WCA) of the PAdaM3 and PAdaM3QA−10% coatings was then measured (Figure 9a,b) and the stability of their temperature-responsive performance was tested. The WCA of the PAdaM3 and PAdaM3QA−10% coatings both drastically increased in the range of 25–30 °C, increasing by more than 30°; the uptrend of the WCA with the increase in the temperature slowed down after 30 °C. As the phase transition of the DMAEMA moiety of the coating fell in the range of 25–30 °C, the hydrophilicity of the coatings was greatly reduced due to the collapse of the hydrophilic segment. In addition, we speculated that the reason for the deviation of the phase transition temperature of the coating and the solution was that the density of the polymer on the surface of the substrate in contact with water was higher than the concentration of the polymer in the solution during the UV-Vis test; thus, the transition temperature of the coating decreased and fell between 25–30 °C. It was notable that the WCA of PAdaM3QA−10% was slightly smaller compared with PAdaM3, indicating that the hydrophilicity was improved after quaternization.

We then tested the WCA of the coatings immersed in water at 25 °C for 30 min and 60 min to evaluate the stability of the coatings. The PAdaM3 and PAdaM3QA−10% coatings both exhibited relatively stable temperature responsiveness. Owing to the host–guest interaction between the β-CD-modified substrate and the adamantyl groups to form a stable inclusion complex in water, the polymer could be stably anchored onto the substrate surface and maintained its temperature-responsive properties.

### 2.7. Antifouling and Antibacterial Properties of PAdaM3QA−10% Coating

We tested the adsorption of bovine serum albumin (BSA) on bare silicon wafers and the PAdaM3QA−10% coating by a fluorescence inverted microscope (FIM) to evaluate the antifouling performance of the coating. The coating was completely immersed in a rhodamine-dyed BSA solution and incubated for 4 h; the silicon wafers were then taken out and washed with a PBS buffer solution at 37 °C or 4 °C, respectively, to remove the unadhered BSA and finally the surface was dried under a stream of dry nitrogen.

**Figure 10:**
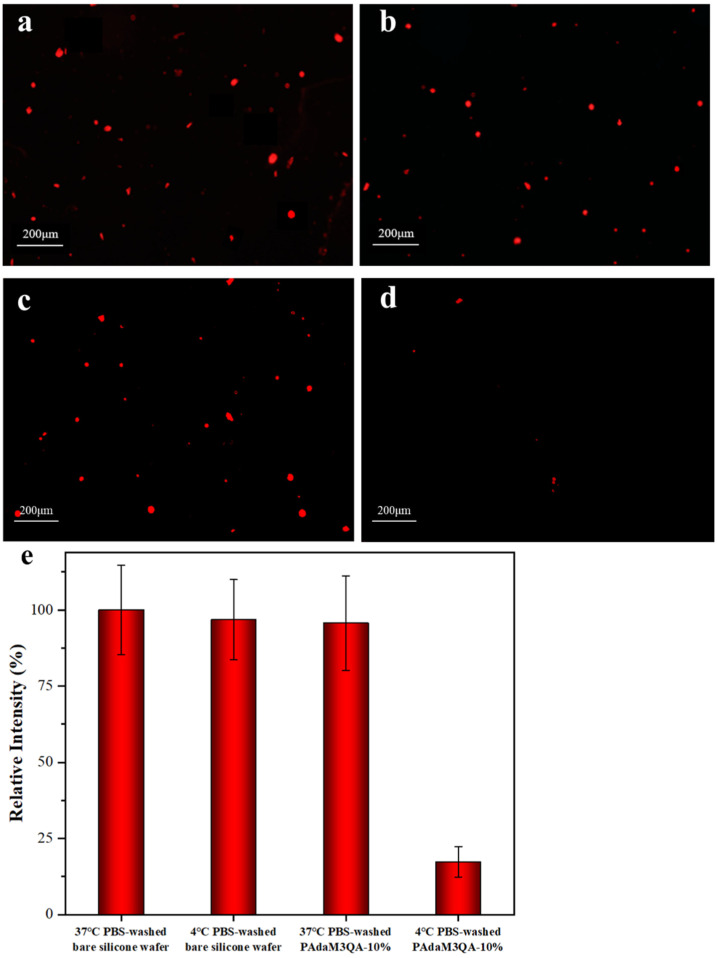
Fluorescent images of BSA adhered on (**a**) 37 °C and (**b**) 4 °C PBS-washed bare silicon wafers, (**c**) 37 °C and (**d**) 4 °C PBS-washed PAdaM3QA−10% coatings, and (**e**) comparison of antifouling performances of bare silicon wafers and PAdaM3QA−10% coatings.

The results of the fluorescence micrographs as shown in Figure 10a–d showed that, compared with the wafers washed with PBS at 37 °C, less BSA adhered to the wafers washed with PBS at 4 °C and the fluorescence intensity of the PAdaM3QA−10%-coated silicon wafers washed with the phosphate buffered saline (PBS) solution at 4 °C decreased by 78.4% (Figure 10d). However, there was no obvious effect on the fluorescence intensity of the bare silicon wafers when they were washed at 37 °C or 4 °C. The mechanism of the release of BSA was attributed to the temperature responsiveness of the PAdaM3QA−10% coatings mentioned in Section 2.5. Upon being rinsed with PBS at 37 °C, the ambient temperature was above the LCST of the coatings (25–30 °C) and the PBS buffer solution contained salt ions, which resulted in the collapse of the DMAEMA moiety. Contrarily, the side chains of PAdaM3QA−10% changed from a collapsed state to an extended state when rinsed with PBS at 4 °C, accompanied by the desorption of BSA at the same time.

**Figure 11:**
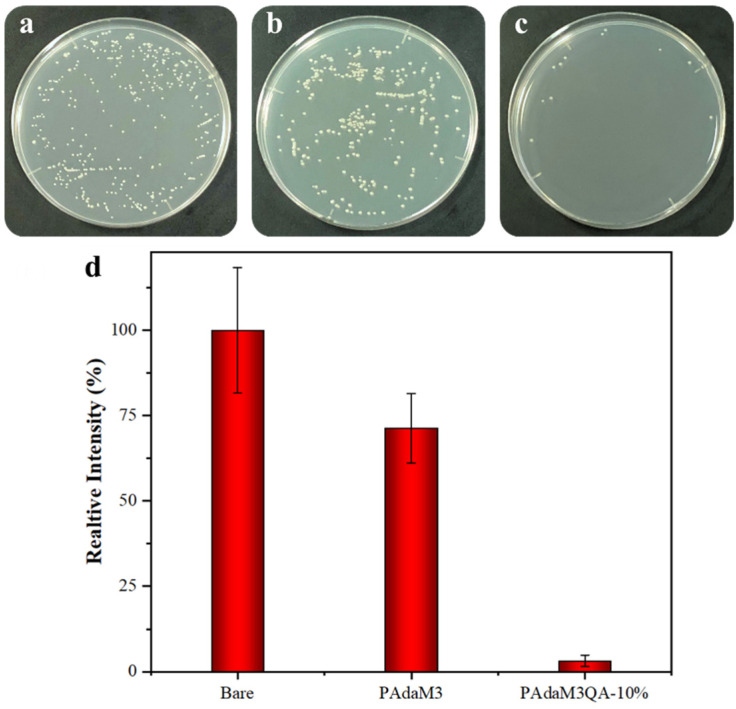
(**a**) Images of bacterial colonies of (**a**) bare, (**b**) PAdaM3, and (**c**) PAdaM3QA−10% coatings; (**d**) comparison of antifouling performances of bare, PAdaM3, and PAdaM3QA−10% coatings.

We evaluated the antibacterial (*E. coli*) property of the PAdaM3QA−10% coating by the spread plate method. After culturing for 24 h, the number of *E* *.coli* colonies on the bare silicon wafers was greater than that of the wafers coated with PAdaM3 and PAdaM3QA−10%, indicating that *E. coli* could easily adhere to the bare silicon wafers (Figure 11a–c). Taking the number of *E* *.coli* colonies on the bare silicon wafers as a reference standard, the sterilization rate of the PAdaM3 coating was only 28.6% whereas the sterilization rate of the PAdaM3QA−10% coating was greatly improved at 96.8% (Figure 11d). The mechanism of the “kill of bacteria” is a result of the electrostatic adsorption between the N^+^ ions generated by the quaternary ammonium groups and the negative charge on the surface of the bacterial cell membrane; the alkane chain connected to the quaternary ammonium group can then penetrate the bacterial cell membrane, thereby destroying the integrity of the bacterial cell membrane, which ultimately leads to the death of bacteria [51,52].

**Figure 12:**
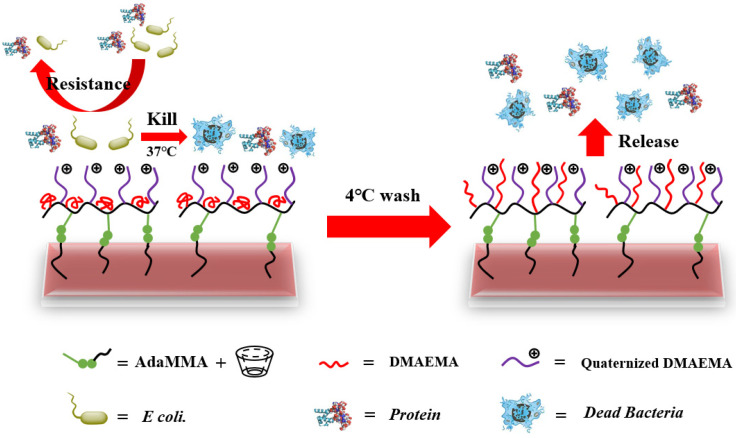
The mechanism of “resistance–kill–release” of PAdaM3QA coating.

According to the results of the WCA, the PAdaM3QA−10% coating had good hydrophilicity as well as good anti-protein adsorption and sterilization properties and could practicably be used in antifouling and antibacterial applications. This could be achieved in three ways (as shown in Figure 12):

(1) The PAdaM3QA coating was hydrophilic at room temperature, which could form a hydration layer on the surface of the coating, thereby requiring more energy for contaminants to break the hydration layer to adhere to the surface and reducing the possibility of the adhesion the of contaminants;
(2) The bacteria could be killed by the quaternary ammonium groups of the PAdaM3QA coating once they adhered to the surface;
(3) The desorption of the adhered contaminants could be achieved by increasing the stretching degree of the tertiary amine groups of the PAdaM3QA coating in water by cooling.

However, as the desorption function of the tertiary amine group and the bactericidal function of the quaternary ammonium group could not be improved at the same time, it is necessary to further study the degree of quaternization corresponding with the best comprehensive antifouling and antibacterial performance.

## 3. Materials and Methods

### 3.1. Materials

Methacryloyl chloride (MAC, ≥95%), 1-adamantane methanol (Ada-OH, ≥99%), 2-(dimethylamino) ethyl methacrylate (DMAEMA, ≥99%), triethylamine (Et_3_N, ≥99.5%), and anisole (≥99.5%) were purchased from Aladdin (Shanghai, China) and used as received. Bromoethane (≥99%), dichloromethane (DCM, ≥99.9%), mono-(6-O-p-methylbenzenesulfonyl)-β-cyclodextrin (≥98%), and tetrahydrofuran (THF, ≥99.5%) were produced by Adamas (Shanghai, China) and used without further treatment. Azo-bis-isobutyronitrile (AIBN, 99%) was supplied by Macklin (Shanghai, China) and recrystallized after being received. HCl (36.5%), NaOH (≥98%), NaCl (≥99.9%), NaBr (≥99%), KI (≥99%), NH_4_Cl (≥99.5%), NaHCO_3_ (≥99.5%), and Na_2_SO_4_ (≥99%) were obtained from Greagent (Shanghai, China). Ultrapure water with a resistivity of 18.25 MΩ cm^−1^ was produced by an ultrapure water purifier system (Milli-Q^®^ IQ 70XX), which was used throughout this study. BSA was purchased from Beijing Biosynthesis Biotechnology Co., Ltd. (Beijing, China). *E. coli* was obtained from Ningbo Mingzhou Biotechnology Co., Ltd. (Ningbo, China).

### 3.2. Preparation of PAdaM3 and PAdaM3QA−10% Coatings

**Figure 13:**
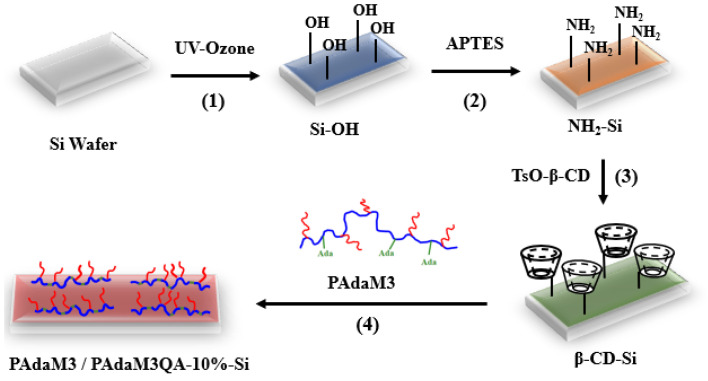
The schematic illustration of PAdaM3/PAdaM3QA−10%-Si.

(1) Pretreatment of silicon wafers (Si-OH in Figure 13): Silicon wafers with a size of 1 cm × 1 cm were ultrasonically cleaned in detergent, acetone, deionized water, and iso-acetone in a sequence for 15 min and dried in an oven. Subsequently, the silicon wafers were treated by UV/ozone for 30 min to hydroxylate the surface.
(2) Preparation of aminated silicon wafers (NH_2_-Si in Figure 13): The hydroxylated silicon wafers were immersed into a mixture of 3-aminopropyltriethoxysilane (APTES) and anhydrous ethanol (*v*/*v* = 1:15), reacted for 12 h at room temperature on an incubation shaker, washed with anhydrous ethanol to remove the unreacted reactants, and dried to obtain the aminated silicon wafers.
(3) Preparation of the β-CD-modified surface (β-CD-Si in Figure 13): mono-(6-O-p-methylbenzenesulfonyl)-β-cyclodextrin (TsO-β-CD) was dissolved in deionized water and the aminated silicon wafer was added to the solution and reacted at 75 °C for 6 h. After the reaction, the wafers were rinsed with deionized water to remove the unreacted reactants to obtain the β-CD-modified wafers.
(4) Preparation of PAdaM3/PAdaM3QA−10%-modified surfaces (PAdaM3/PAdaM3QA−10%-Si in Figure 13): PAdaM3 or PAdaM3QA−10% was dissolved in deionized water, β-CD-Si was immersed in the solution and reacted at room temperature for 24 h. After being rinsed with deionized water and dried, a PAdaM3/PAdaM3QA−10%-modified surface was obtained.

### 3.3. UV-Vis Spectrometer Test

The transmittance of the polymer aqueous solution was measured by a UV-Vis spectrophotometer of PerkinElmer Lambda 750S. The temperature of the solutions was controlled by a Brookfield circulating water bath temperature controller. The solution of the samples was loaded into a 1.0 cm × 1.0 cm quartz and heated in a water bath; the heating or cooling rate of the water bath was 1 °C/min. The cloud point of the solutions was defined as the temperature at which the transmittance dropped to 50%. A 500 nm wavelength was applied in the test.

### 3.4. Water Contact Angle (WCA) Test

The contact angle of the water on the surface at different temperatures was measured by a DS100 contact angle meter from the KRUSS Company, Germany. About 5 µL of water droplets were injected for the test and the system used the Laplace–Young fitting algorithm to automatically calculate the water contact angle.

### 3.5. Zeta Potential Test

The solution of the samples was loaded into a 1.0 cm × 1.0 cm quartz. The zeta potential of the polymer aqueous solutions at different pHs was measured by a ZS90 nanoparticle size and zeta potential analyzer from Malvern, UK.

### 3.6. Anti-Protein Adhesion Test

The protein adhesion resistance of the polymer coatings was evaluated by testing the fluorescence intensity of the BSA adsorbed on the surface. A total of 1.0 mg/mL BSA was dissolved in a PBS buffer as the test solution; the silicon wafers were immersed in the solution and incubated for 4 h. Subsequently, the wafers were repeatedly rinsed with a PBS buffer solution to remove the BSA that had not adhered to the coating and were dried under a nitrogen flow. The surface was observed using an inverted fluorescence microscope and the relative fluorescence intensity images of the surface were recorded. The fluorescence intensity was quantitatively analyzed using ImageJ to evaluate the adhesion of the BSA.

### 3.7. Antibacterial Test

The antibacterial properties of the polymer coatings were tested by *E. coli* (ATCC-700926). First, *E. coli* was incubated in a Luria–Bertani broth medium at 37 °C for 12 h. The liquid medium was subsequently removed by centrifugation and then washed 3 times with sterile PBS (pH = 7.4). Finally, the PBS solution of *E. coli* was diluted to 1 × 10^7^ CFU/mL as the bacterial suspension for testing.

The PAdaM3QA−10% coating was sterilized with 75% ethanol and washed 3 times with PBS to remove the 75% ethanol. The surface was then completely dried for use. The above-prepared bacterial suspension (10 μL, 1 × 10^7^ CFU/mL) was dropped on the middle area of the PAdaM3QA−10% coating and covered with a PE film, followed by incubation at 37 °C for 24 h. The silicon wafers were then transferred to a sterile test tube containing 2 mL of the PBS buffer. After sonication for 5 min, the bacterial suspension was diluted and 200 μL of the bacterial suspension was evenly spread onto the solid medium. The number of bacterial colonies was recorded after incubation at 37 °C for 24 h to evaluate the antibacterial performance of the PAdaM3QA−10%-coated surface.

## 4. Conclusions

The introduction of AdaMMA reduced the number of hydrogen bonds between the copolymer and water, resulting in an obvious decrease in the LCST with an increase in the amount of AdaMMA added. The LCST also increased as the degree of quaternization increased and ultimately disappeared when the degree of quaternization exceeded 20%.

PAdaMX and PAdaM3QA−10% both lost their temperature-responsive performance due to the protonation of side chains when the pH was less than the pK_a_; however, when it was in an alkaline environment, the LCST decreased approximately linearly with the increase in the pH because of the increase in the hydrophobic interaction. In addition, PAdaM3QA−10% was more sensitive to the pH changes than PAdaM3 because of the different pH sensitivity of the quaternary and tertiary amine groups. The electrostatic screening of ions weakened the intramolecular mutual repulsion, resulting in a decrease in the LCST; the ability of different ions to decrease the LCST was similar to the Hoffman sequence. Moreover, PAdaM3QA−10% was less sensitive to the change in ion concentration due to the stronger intramolecular mutual repulsion of the quaternary amine groups. β-CD could increase the hydrophilicity of the polymer via a host–guest interaction with the adamantyl groups in the polymers, which increased the LCST. The adamantyl groups in the polymers served as an anchoring moiety to the substrate, enabling the polymers to stably bind to the surface.

The enhanced hydrophilicity of PAdaM3QA−10% could help to form a hydration layer to resist contaminants to adsorb on the surface. The function of antifouling was achieved by the “release” mechanism of the temperature-responsive tertiary amine groups. The antibacterial function was realized by the quaternary amine groups. In summary, the “resistance–kill–release” mechanism for antifouling and antibacterial functions was achieved by the preparation of stimuli-responsive amphiphilic copolymer PAdaM3QA−10%.

## Figures and Tables

**Table 1:** The summary of the structure of PAdaMX with different [AdaMMA]:[DMAEMA] feed ratios.

Sample	Feeding [AdaMMA]: [DMAEMA]	Monomer Consumption	Product [AdaMMA]: [DMAEMA]	Mn (g/mol) (THF Phase)	M_w_/M_n_
PAdaM0	0:100	96.64%	0:100	24,840	2.797
PAdaM1	1:99	99.16%	1.15:98.85	20,060	2.653
PAdaM3	3:97	98.40%	2.91:97.09	20,958	2.518
PAdaM5	5:95	96.78%	5.52:94.48	19,839	2.548

**Table 2:** The degree of quaternization of PAdaM3QA−X.

Sample	Estimated Degree of Quaternization	S_3.20_ (Normalized)	S_2.30_	Actual Degree of Quaternization
PAdaMAQA-5%	5%	6.0	101.12	5.6%
PAdaMAQA-10%	10%	6.0	45.16	11.7%
PAdaMAQA-20%	20%	6.0	18.29	24.7%
PAdaMAQA-50%	50%	6.0	5.18	53.7%
PAdaMAQA-80%	80%	6.0	1.56	79.4%
PAdaMAQA-100%	100%	6.0	0.00	100.0%

**Table 3:** The parameters of the fitting curves for LCST ion concentrations of PAdaM3 and PAdaM3QA−10% solutions.

Sample	Salt	Slope (*k)*	Intercept (*b*)	Correlation Coefficient (*R*^2^)
PAdaM3	NaCl	−2.42	32.68	0.972
	NaBr	−1.75	32.87	0.982
	KI	−1.30	32.90	0.963
PAdaM3QA−10%	NaCl	−2.12	38.26	0.995
	NaBr	−1.72	38.30	0.988
	KI	−1.07	38.30	0.986

## Data Availability

The data presented in this study are available on request from the corresponding author.

## Sample Availability

Samples of PAdaMX and PAdaM3QA−X are available from the authors.

## References

[1] Kwon I.C., Bae Y.H. (1991). Electrically erodible polymer gel for controlled release of drugs. Nature.

[2] Suzuki A., Tanaka T. (1990). Phase transition in polymer gels induced by visible light. Nature.

[3] Gutowska A., Bae Y.H. (1994). Thermosensitive interpenetrating polymer networks: Synthesis, characterization, and macromolecular release. Macromolecules.

[4] Sijbesma R.P., Beijer F.H. (1997). Reversible polymers formed from self-complementary monomers using quadruple hydrogen bonding. Science.

[5] Zou S., Ma Y.J. (2004). Grafting of single, stimuli-responsive poly (ferrocenylsilane) polymer chains to gold surfaces. Langmuir.

[6] Okada S., Sato E. (2021). Thermo- and photo- responsive behaviors of dual-stimuli-responsive organogels consisting of homopolymers of coumarin-containing methacrylate. Polymers.

[7] Thomas C.S., Xu L. (2012). Kinetically controlled nanostructure formation in self-assembled globular protein-polymer diblock copolymers. Biomacromolecules.

[8] Nash M.A., Waitumbi J.N. (2012). Multiplexed enrichment and detection of malarial biomarkers using a stimuli-responsive iron oxide and gold nanoparticle reagent system. ACS Nano.

[9] Ayano E., Karaki M. (2012). Poly (N-isopropylacrylamide)–PLA and PLA blend nanoparticles for temperature-controllable drug release and intracellular uptake. Colloid Surf. B.

[10] Kawamura A., Harada A. (2021). Weakly acidic pH and reduction dual stimuli-responsive gel particles. Langmuir.

[11] Asadi-Zaki N., Mardani H. (2021). Interparticle cycloaddition reactions for morphology transition of coumarin-functionalized stimuli-responsive polymer nanoparticles prepared by surfactant-free dispersion polymerization. Polymers.

[12] Li Z.H., Yang H.L. (2021). Theoretical and experimental insights into the self-assembly and ion response mechanisms of tripodal quinolinamido-based supramolecular organogels. ChemPlusChem.

[13] Wang L., Liu M. (2010). A pH-, thermo-, and glucose-, triple-responsive hydrogels: Synthesis and controlled drug delivery. React. Funct. Polym..

[14] Ouyang H., Xia Z. (2010). Voltage-controlled flow regulating in nanofluidic channels with charged polymer brushes. Microfluid. Nanofluid..

[15] Kanazawa H., Okano T. (2011). Temperature-responsive chromatography for the separation of biomolecules. J. Chromatogr. A.

[16] Nagase K., Kobayashi J. (2008). Influence of graft interface polarity on hydration/dehydration of grafted thermoresponsive polymer brushes and steroid separation using all-aqueous chromatography. Langmuir.

[17] Liu C.F., Lin H. (2022). Smart responsive photoelectric organic modulator integrated with versatile optoelectronic characteristics. Adv. Funct. Mater..

[18] Hou Z.S., Sun Y.L. (2020). Smart bio-gel optofluidic Mach-Zehnder interferometers multiphoton-lithographically customized with chemo-mechanical-opto transduction and bio-triggered degradation. Lab Chip.

[19] Williams L., Hatton F.L. (2022). Electrospinning of stimuli-responsive polymers for controlled drug delivery: pH- and temperature-driven release. Biotechnol. Bioeng..

[20] Hershberger K.K., Gauger A.J. (2021). Utilizing stimuli responsive linkages to engineer and enhance polymer nanoparticle-based drug delivery platforms. ACS Appl. Bio Mater..

[21] Wang T.Y., Tsao H.K. (2020). Perforated vesicles of ABA triblock copolymers with ON/OFF-switchable nanopores. Macromolecules.

[22] Chang W.H., Lee Y.F. (2021). Stimuli-responsive hydrogel microcapsules for the amplified detection of microRNAs. Nanoscales.

[23] Reese C.J., Qi Y.R. (2022). Antifouling oligoethylene glycol-functionalized aromatic polyamides brushes synthesized via surface-initiated chain-growth condensation polymerization. ACS Appl. Polym. Mater..

[24] Chiarcos R., Antonioli D. (2022). Short vs. long chains competition during “grafting to” process from melt. Polym. Chem..

[25] Aboudzadeh M.A., Kruse J. (2021). Gold nanoparticles endowed with low-temperature colloidal stability by cyclic polyethylene glycol in ethanol. Soft Matter.

[26] Ye H., Zhou Y.N. (2022). Protein fractionation of Ph-responsive brush-modified ethylene vinyl alcohol copolymer membranes. Polym. Eng. Sci..

[27] Parsonage E., Tirrell M. (1991). Adsorption of poly (2-vinylpyridine)-poly (styrene) block copolymers from toluene solutions. Macromolecules.

[28] Marra J., Hair M.L. (1989). Interactions between two adsorbed layers of poly (ethylene oxide)/polystyrene diblock copolymers in heptane-toluene mixtures. Colloids Surf..

[29] Liu X., Appelhans D. (2018). Hollow capsules with multiresponsive valves for controlled enzymatic reactions. J. Am. Chem. Soc..

[30] Karakasyan C., Sebille B. (2007). The reversible binding of anti-human serum albumin to poly beta-cyclodextrin-coated porous silica supports. J. Chromatogr. B.

[31] Yang W.J., Neoh K.G. (2014). Polymer brush coatings for combating marine biofouling. Prog. Polym. Sci..

[32] Neoh K.G., Kang E.T. (2011). Combating bacterial colonization on metals via polymer coatings: Relevance to marine and medical applications. ACS Appl. Mater. Interfaces.

[33] Stamou A., Iatrou H. (2022). NIPAm-based modification of poly(L-lysine): A pH-dependent LCST-type thermo-responsive biodegradable polymer. Polymers.

[34] Gungor Z., Ozay H. (2022). Synthesis of new type temperature and pH sensitive hydrogels using drug-based p-(methacryloyloxy)acetanilide monomer and their usage as controlled drug carrier material. J. Macromol. Sci. Part A-Pure Appl. Chem..

[35] Wei T., Yu Q. (2019). Responsive and synergistic antibacterial coatings: Fighting against bacteria in a smart and effective way. Adv. Healthc. Mater..

[36] Ilčíková M., Tkáč J. (2015). Switchable materials containing polyzwitterion moieties. Polymers.

[37] Cheng G., Xue H. (2008). A switchable biocompatible polymer surface with self-sterilizing and nonfouling capabilities. Angew. Chem. Int. Ed..

[38] Cao Z., Mi L. (2012). Reversibly switching the function of a surface between attacking and defending against bacteria. Angew. Chem. Int. Ed..

[39] Li X., Wu B. (2018). Recent developments in smart antibacterial surfaces to inhibit biofilm formation and bacterial infections. J. Mat. Chem. B.

[40] Ding X., Duan S. (2018). Versatile antibacterial materials: An emerging arsenal for combatting bacterial pathogens. Adv. Funct. Mater..

[41] Xia Y.Q., Adibnia V. (2019). Biomimetic bottlebrush polymer coatings for fabrication of ultralow fouling surfaces. Angew. Chem.-Int. Edit..

[42] Cho S.H., Jhon M.S. (1997). Temperature-induced phase transition of poly(N,N-dimethylaminoethyl methacrylate-co-acrylamide). J. Polym. Sci. Part B-Polym. Phys..

[43] Wang B.L., Ye Z. (2016). Construction of a temperature-responsive terpolymer coating with recyclable bactericidal and self-cleaning antimicrobial properties. Biomater. Sci..

[44] Gamnino S.R. (1963). The value of “pK_1_” in the Henderson-Hasselbalch equation. Scand. J. Clin. Lab. Invest.

[45] Zhang H., Marmin T. (2015). A new comonomer design for enhancing the pH-triggered LCST shift of thermosensitive polymers. Polym. Chem..

[46] Liu F., Urban M.W. (2008). Dual temperature and pH responsiveness of poly(2-(N,N-dimethylamino)ethyl methacrylate-co-n-butyl acrylate) colloidal dispersions and their films. Macromolecules.

[47] Baldwin R.L. (1996). How Hofmeister ion interactions affect protein stability. Biophys. J..

[48] Zhang Y., Furyk S. (2005). Specific ion effects on the water solubility of macromolecules: PNIPAM and the Hofmeister series. J. Am. Chem. Soc..

[49] Munteanu M., Choi S. (2008). Supramolecular structures based on dimeric combinations of cyclodextrin and adamantane via click chemistry. J. Incl. Phenom. Macrocycl. Chem..

[50] Chali S.P., Azhdari S. (2021). Biodegradable supramolecular micelles via host-guest interaction of cyclodextrin-terminated polypeptides and adamantane-terminated polycaprolactones. Chem. Commun..

[51] Jennings M.C., Minbiole K.P.C. (2015). Quaternary ammonium compounds: An antimicrobial mainstay and platform for innovation to address bacterial resistance. ACS Infect. Dis..

[52] Denyer S.P. (1995). Mechanisms of action of antibacterial biocides. Int. Biodeterior. Biodegrad..

